# Ontology based text mining of gene-phenotype associations: application to candidate gene prediction

**DOI:** 10.1093/database/baz019

**Published:** 2019-02-27

**Authors:** Şenay Kafkas, Robert Hoehndorf

**Affiliations:** Computer, Electrical and Mathematical Sciences & Engineering Division, Computational Bioscience Research Center, King Abdullah University of Science and Technology, Thuwal, Kingdom of Saudi Arabia

## Abstract

Gene–phenotype associations play an important role in understanding the disease mechanisms which is a requirement for treatment development. A portion of gene–phenotype associations are observed mainly experimentally and made publicly available through several standard resources such as MGI. However, there is still a vast amount of gene–phenotype associations buried in the biomedical literature. Given the large amount of literature data, we need automated text mining tools to alleviate the burden in manual curation of gene–phenotype associations and to develop comprehensive resources. In this study, we present an ontology-based approach in combination with statistical methods to text mine gene–phenotype associations from the literature. Our method achieved AUC values of 0.90 and 0.75 in recovering known gene–phenotype associations from HPO and MGI respectively. We posit that candidate genes and their relevant diseases should be expressed with similar phenotypes in publications. Thus, we demonstrate the utility of our approach by predicting disease candidate genes based on the semantic similarities of phenotypes associated with genes and diseases. To the best of our knowledge, this is the first study using an ontology based approach to extract gene–phenotype associations from the literature. We evaluated our disease candidate prediction model on the gene–disease associations from MGI. Our model achieved AUC values of 0.90 and 0.87 on OMIM (human) and MGI (mouse) datasets of gene–disease associations respectively. Our manual analysis on the text mined data revealed that our method can accurately extract gene–phenotype associations which are not currently covered by the existing public gene–phenotype resources. Overall, results indicate that our method can precisely extract known as well as new gene–phenotype associations from literature. All the data and methods are available at https://github.com/bio-ontology-research-group/genepheno.

## Introduction

Phenotypes are the observable characteristics of an organism resulting from its genotype and response to environment. Associations of genotypes and phenotypes shed light on our understanding of disease mechanisms as they provide a way of observing the indirect consequences of multi-scale physiological interactions occurring within an organism.

Phenotypes are recorded in the context of human genetics as well as in animal model experiments, and are made available in clinical databases such as ClinVar (1), Online Mendelian Inheritance in Men (OMIM) (2), or the Human Phenotype Ontology (HPO) (3).

The diversity of phenotypes makes it challenging to represent them in a way that is comparable within and across databases. In response to this challenge, phenotype ontologies have been developed that formally represent phenotypes in several species and enable their integration and comparison (4). While the majority of phenotype ontologies was species-specific and limited to one – or a few related – species, there has been significant effort in integrating phenotype ontologies recently so that phenotypes across species can be compared and jointly analyzed (5–7).

One of the successful applications of computational representation and integration of phenotypes across species is the prioritization of candidate genes for disease (7, 8) as well as identification of causative variants in personal genome sequences (9–11). These applications rely on a database of associations between a gene and a set of phenotypes coming either from human clinical observations, or from model organisms such as the Mouse Genome Informatics (MGI) database (12), and comparing disease or patient phenotypes to this database of gene–phenotype associations.

While high-throughput phenotyping studies such as those performed by the International Mouse Phenotyping Consortium (IMPC) (13) can automatically generate formal, ontology-based phenotype descriptions and deposit them in public databases, a large number of phenotyping experiments are primarily reported in literature. Consequently, literature curation remains as one of the main sources of phenotype information and is widely applied in model organism databases such as the MGI database. With an increasing number of experimental results and publications, literature curation alone is faced with challenges in providing accurate and recent data.

Here, we describe a text mining system to extract associations between human and mouse genes and their phenotypes. Our text mining method relies on identifying mentions of genes or proteins and mentions of phenotypes from HPO and the Mammalian Phenotype Ontology (MP) (14) ontologies in text. We further utilize these ontologies as background knowledge during text mining to increase the coverage of annotations that are not explicitly mentioned in text but rather implied based on the semantics in the ontologies. We then use normalized pointwise mutual information (NPMI) (15, 16) on this enriched information to measure the strength of gene–phenotype associations. We evaluate the phenotypes by comparing them to known gene–phenotype associations available from reference databases and we demonstrate that the gene–phenotype associations we extract can improve prioritization of disease genes based on phenotype similarity between genes and diseases.

## Results

### Ontology-based mining of gene–phenotype associations

We developed a method to mine gene–phenotype associations from the literature, using the knowledge contained in the phenotype ontologies as background knowledge. We use the WhatIzIt (17) named entity recognition and normalization tool to recognize gene or protein mentions and normalize them to the UniProt/Swiss-Prot database (18), and we identify phenotype mentions and normalize them to two phenotype ontologies, MP and HPO. While recognition of gene and protein names in literature is a well-established task for which several mature methods exist (19, 20), and for which WhatIzIt is known to perform competitively (21), recognizing phenotype mentions in literature is challenging because their descriptions are both syntactically and semantically complex due to the high heterogeneity of phenotypes (4). Furthermore, phenotypes are organized in ontologies in a class hierarchy that is generated based on axioms used to constrain phenotype classes (22). While the use of the axiomatic information in ontologies as background knowledge has the potential to improve the performance and robustness of text mining approaches (23), it also increases the complexity of the task.

We use a phenotype ontology as background knowledge when determining which phenotype is mentioned in a particular location in literature. Specifically, we assume that, if *P_1_* is a subclass of *P_2_* in a phenotype ontology *O*, then all mentions of *P_1_* are also mentions of *P_2_* (with respect to *O*). For example, *Central Nervous System (CNS) inflammation* (MP:0006082) has the subclass *Brain inflammation* (MP:0001847) in MP; *Brain inflammation* is further inferred to be equivalent to the class *Encephalitis* (HP:0002383) in the PhenomeNET ontology (7). We use these axioms to construct the set of terms that refer to *CNS inflammation* as the set consisting of `CNS inflammation', `Brain inflammation', and `Encephalitis'.

The aim of using the axioms of the ontology as background knowledge is to propagate information about which terms may be used to refer to a phenotype over the ontology hierarchy, thereby extend the set of strings that refer to a phenotype and make our text mining approach more robust. Furthermore, the propagation of information over the ontology hierarchy allows us to test for significant association between a gene and phenotype on all levels of the phenotype ontology, therefore improving robustness of our statistical approach.

To determine whether a gene or protein and a phenotype are associated, and to determine the strength of the association, we first measure co-occurrence of gene and phenotype mentions in sentences within a corpus, and we then use a statistical measure that determines the strength of a co-occurrence. The measure assumes that significantly co-mentioned genes and phenotypes stand in a biological relation.

We apply our method to all of the full text articles in the PubMed Central corpus of Open Access articles. The corpus consists of 1 596 360 full text articles. Within this corpus, we identified a total number of 571 980 articles which contain both the mention of a gene/protein and a phenotype within a sentence. These contain 4 665 170 co-mentions between 16 860 genes (15 928 of them have reference to both MGI and the Entrez Gene Database while the remaining 932 have reference only to the Entrez Gene Database) and 11 097 phenotype of which 5182 and 5915 are from MP and HP classes.

We do not distinguish between gene and protein mentions due to well-known difficulties in disambiguating between them (24). Furthermore, we do not distinguish between different species in which a gene or protein is found. Therefore, we combine the human and mouse gene/protein names and identify proteins by either their human or mouse gene identifier in the Entrez Gene Database (treating human–mouse orthologs as equivalent).

We score each association between a gene and phenotype using the NPMI (15, 16) measure. While NPMI is commonly a measure of co-occurrence strength between two terms, we extend NPMI to measure the co-occurrence strength between a class of genes or proteins and a class from a phenotype ontology *O*, considering the background knowledge in *O*. For this purpose, we identify, for every class, the set of labels and synonyms associated with the class (*Labels(C)* denotes the set of labels and synonyms of *C*). We then define *Terms(C)* as the set of all terms that can be used to refer to *C*: *Terms(C)*: = $ \{\
\textit{x} \vert \textit{x} \in \textit{Labels} (\textit{S})
\wedge{S} \sqsubseteq C\,\} $ i.e., we consider all terms referring to either *C* or any of *C*’s subclasses as referring to *C*. Then, we calculate the NPMI between a gene *G* and a class *D* as

(1) \begin{equation*} npmi(G,D)=\frac{\textrm{log}\frac{n_{G,D}\cdot n_{tot}}{n_{G}\cdot n_{D}}}{-\textrm{log}\frac{n_{G,D}}{n_{tot}}} \end{equation*}

where *n_tot_* is the total number of sentences in our corpus, *n_G,D_* is the number of sentences in which both a mention of G and a term from *Terms(D)* co-occur, *n_G_* is the number of sentences in which a mention of *G* occurs, and *n_D_* is the number of sentences in which a term from *Terms(D)* occurs.

### Determining rank threshold

Each of the gene–phenotype associations is scored by an NPMI value that measures the strength of the association. Using this NPMI value, we rank phenotypes for each gene. The next step in our method is to determine a threshold for a significant association, and for this purpose we determine the similarity between our text-mined phenotypes and experimentally determined and manually curated phenotype annotations. Specifically, we change the threshold rank for considering phenotypes as associated with a gene (i.e., we consider only the top *n* ranked phenotypes for each gene as associated, with varying *n*), and we optimize the predictive performance when using these phenotypes in finding manually annotated genes in two different databases of gene–phenotype associations; the underlying assumption of this test is that the text-mined phenotypes should be as close as possible to the manually curated phenotypes. We used two datasets for comparison, one of human gene–phenotype associations observed in a clinical context and represented in the HPO database (3), and another of mouse gene–phenotype associations coming from mouse model studies and represented in the Mouse Genome Informatics (MGI) (12) database. We used Resnik’s semantic similarity measure (25) for comparison which is one of the most widely used semantic similarity measurements in life sciences (26, 27), and we combine the pairwise phenotype–phenotype similarity using the Best Match Average (BMA) strategy to measure the phenotypic similarities of genes. We use the PhenomeNET ontology (7) to compute semantic similarity as it integrates the MP and HPO ontologies (28) and therefore enables computation of phenotype similarity irrespective of which ontology is used to characterize a phenotype or in which species (human or mouse) a phenotype has been observed.

**Figure 1:**
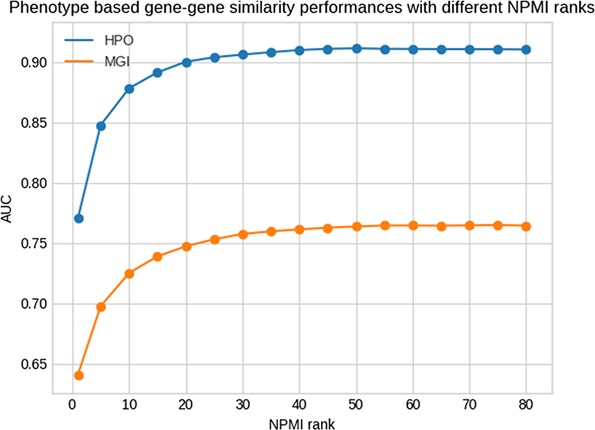
Phenotype-based gene–gene similarity with different NPMI ranks.

Figure 1 shows the results of this test for different NPMI ranks. The performance of retrieving the same genes by phenotype similarity is higher when using human phenotypes compared to mouse phenotypes, indicating that the phenotypes extracted from the literature are more similar to human gene phenotypes rather than the mouse gene phenotypes. Our method achieves the best performance at the rank of 50 and 75 for the HPO and MGI datasets measured using the area under the receiver operating characteristic curve (AUC) (29), with AUC values of 0.91 and 0.77, respectively. However, for our analysis we use the threshold of 25 with AUC values of 0.90 and 0.75 for HPO and MGI to have a smaller set of phenotypes with less potential for false positives.

**Table 1:** Distribution of gene-phenotype associations in text mined and reference datasets

Dataset	Number of gene-phenotype associations
literature (HPO + MGI)	295 971
literature (HPO)	206 156
literature (MGI)	189 815
Reference (HPO + MGI)	300 345
Reference (HPO)	99 333
Reference (MGI)	211 012
Intersection between literature and Reference (HPO + MGI)	11 473
Intersection between literature (HPO) and Reference (HPO)	5650
Intersection between literature (MGI) and Reference (MGI)	5821

Table 1 shows the statistics of the number of gene–phenotype associations we obtain through text mining at the rank threshold of 25. There are a total of 395 971 and 300 345 gene–phenotype associations in the literature and reference datasets (MGI and HPO combined). Further analysis on the two sets shows that only 11 473 (1.7% of whole set) of the gene–phenotype associations directly overlap (i.e., without considering inheritance using the ontology structure). Combined with the high similarity to known associations, this analysis shows that the text-mined phenotypes are often related but more or less specific than the phenotypes included in curated databases; in most cases, we will associate a more general class while literature curation can identify more specific classes, due to our statistical approach in which we propagate information over the ontology structure and therefore often find more general associations. Furthermore, our literature-based approach also identifies novel associations that are not yet included in the curated databases.

**Figure 2:**
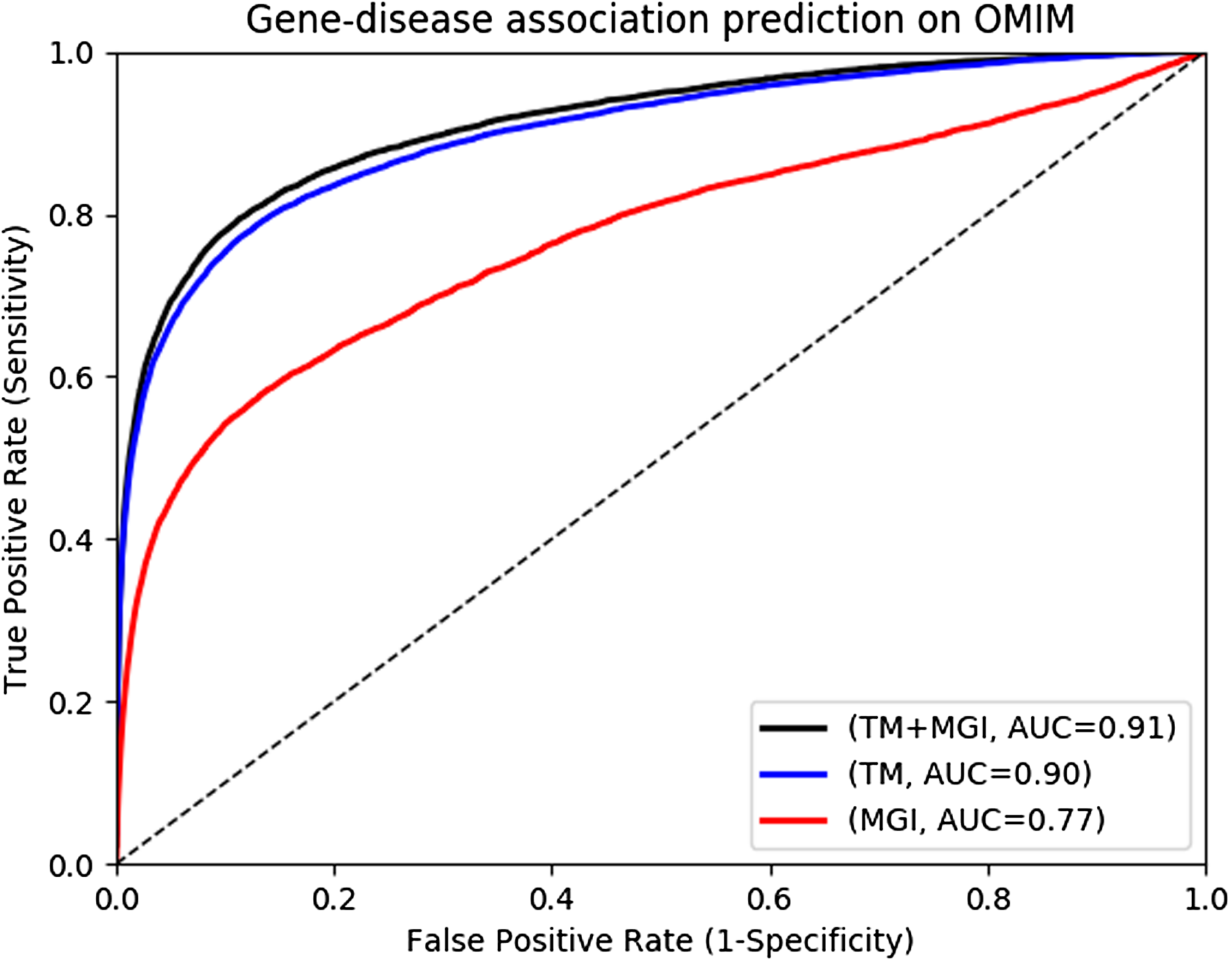
Gene-disease association prediction performances on OMIM.

**Figure 3:**
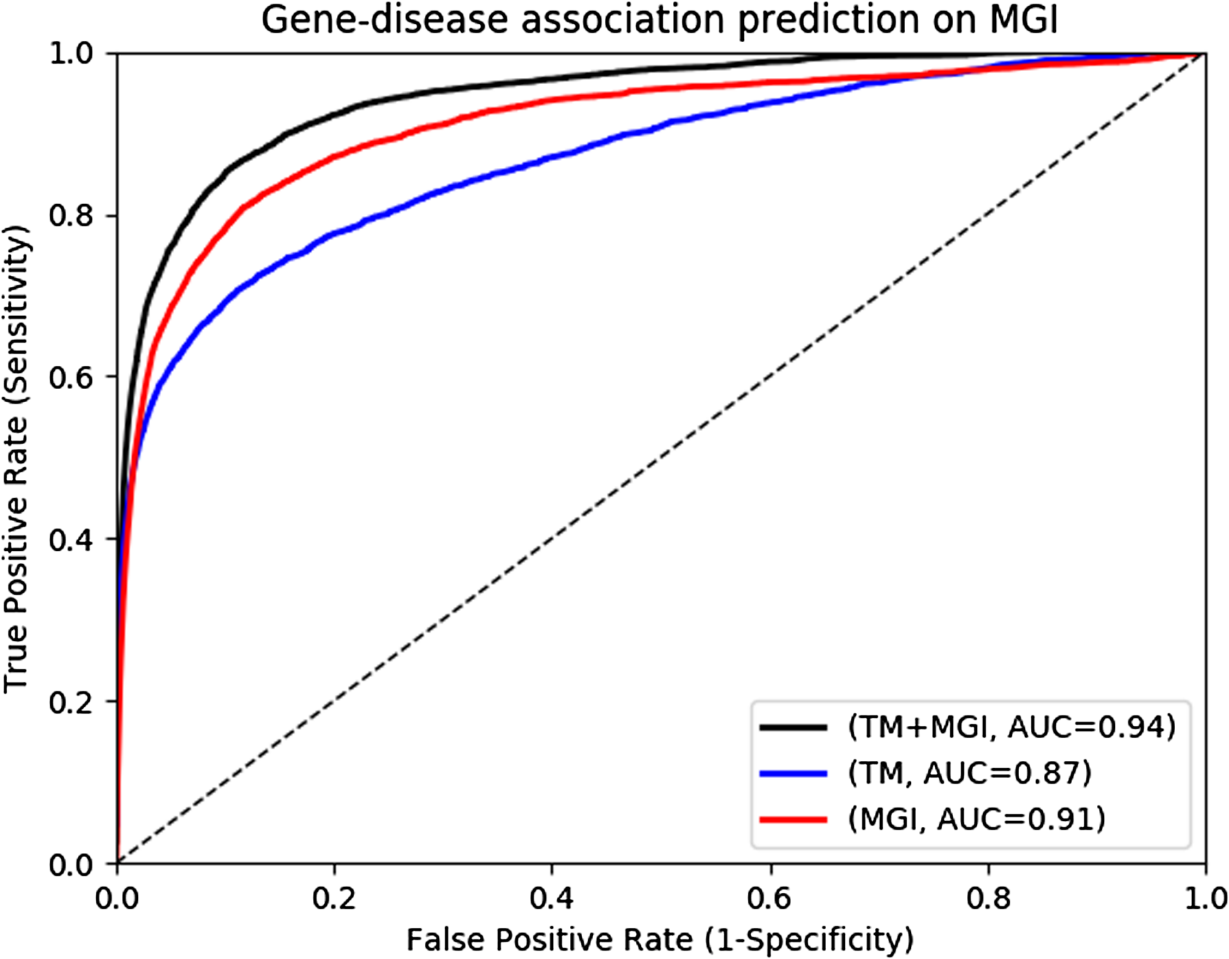
Gene-disease association prediction performances on MGI.

### Text-mined phenotypes recover disease genes

As external evaluation of our text mining method, we utilize our text mined gene-phenotype associations and disease-phenotype associations gathered from HPO and predict gene–disease associations based on the semantic similarity of phenotypes linked to genes and diseases. We used the PhenomeNET ontology (7) as reference ontology for similarity computation as it allows integration and comparison of phenotypes in multiple species, and we evaluate the predictions on clinical gene–disease associations from the OMIM database as well as a set of mouse models of human disease from MGI.

The MGI and HPO contain a total of 12 063 and 3738 genes which have phenotype associations, respectively. Through our text mining approach we associate a total of 16 808 human genes with phenotypes and can use them for phenotype-based prediction of gene–disease associations. Figure 2 and Figure 3 show our gene–disease association prediction performances on OMIM and MGI respectively. Using text mined gene–phenotype associations to predict gene–disease associations results in an AUC of 0.90 using human gene–disease associations from OMIM and 0.87 when identifying mouse models of human disease from MGI. On the other hand, use of the curated gene–phenotype associations from MGI to predict gene–disease associations yields an AUC of 0.77 for OMIM and 0.91 for mouse models of human disease. When text-mined and experimentally validated gene–phenotypes are combined, the prediction performance further increases to 0.91 and 0.94 on OMIM and MGI, respectively, demonstrating that text-mined and experimentally validated phenotypes contain complementary information.

## Discussion

To the best of our knowledge, we present the first ontology-based text mining system for extracting gene–phenotype associations from the literature, in particular for human and mouse. Previously, several text-mining systems for extracting gene–phenotype associations were developed (30–33). One approach is based on unsupervised learning that combines text mining with comparative genome analysis to associate genes and their phenotypic characteristics (30). In this approach, first they annotate terms reflecting phenotypic similarities of species in text and then they identify gene–phenotype associations systematically based on the similarity of their phyletic distribution; they do not use controlled vocabularies or ontologies in their work. Another approach relies on machine learning methods to extract the gene–phenotype associations from text (31). This study focuses on a small number (ten) disease concepts from the Medical Subjects Headings (MeSH) (34) terminology as phenotypes. Khordad and Mercer proposes a semi-supervised learning method to identify genotype–phenotype relationships from biomedical text (32). The method starts with semi-automatically creating a seed set of labeled data from an unlabeled genotype–phenotype dataset and applying named entity recognition tools to annotate the dataset, manually curate it, and then training a machine learning model using the seed data to identify gene–phenotype relations in text. Xing et. al is using unsupervised machine learning methods to extract gene–phenotype relations from text and apply the methods to plant phenotypes (33). This system relies on a combination of rule-based and lexical methods to identify plant gene names, and an unsupervised representation learning approach to identify plant phenotypes in text. While all these approaches also target the extraction of gene–phenotype associations from literature, there are several shared differences to our approach. First, none of the previous approaches are evaluated with respect to their utility for predicting gene–disease associations, while our main focus is to identify the associations that can predict these associations based on comparison with experiment data contained in model organism databases. Second, none of the previous approaches utilize ontologies as background knowledge during the text mining. Third, we focus specifically on extraction of phenotypes that are associated with human and mouse genes while other approaches target different organisms. Finally, while we consider all the phenotype classes from the HP and MP ontologies, most studies do not consider ontology-based representations of phenotypes but rather use terminologies such as MeSH (31).

The methods as well as the data presented in this study can be further utilized in data and text mining workflows, and our method is generic and can be applied to extract associations between other biomedical entities from the literature when entities use ontology classes (35).

Currently, there are gene–phenotype associations for around 50% of protein-coding genes in the mouse (there are 12 063 protein-coding genes out of 24 408 in MGI with phenotype associations). Many of these phenotype associations come from high-throughput phenotyping experiments such as those performed as part of the International Mouse Phenotyping Consortium (IMPC) (13) while others are based on literature curation (12). Our approach is mainly aimed at helping curators to identify phenotype associations for inclusion in a phenotype database as well as to provide a large set of computationally generated gene–phenotype associations that are not yet included in MGI or similar databases and which can be used for computational analyses. While our approach cannot match the accuracy and depth of annotation that can be achieved by a curator, the computationally generated gene–phenotype associations can nevertheless be of use for computational studies. We have demonstrated their utility by applying them to recover gene–disease associations based on phenotype similarity, and demonstrate that our approach can improve predictive performance.

Importantly, our approach can also suggest phenotype associations for genes which have no associated pheno-types in a phenotype database yet. For example, we identify an association between the *Icam5* (MGI:109430, ENTREZ:7087) gene and *Encephalitis* (HP:0002383) based on our method, while there are no phenotypes associated with *Icam5* in the HPO database. Similarly, the association between *Pnma2* (MGI:2444129, ENTREZ:10687) and *encephalitis* (HP:0002383) is not included in either MGI or the HPO database although recent evidence suggests such an involvement (PMID:27003254) (36).

Our approach has some limitations given that text mining results often contain both false positives (samples which are wrongly annotated as positive class) and false negatives (missed annotations). In our extracts, we observed some false negatives due to failure to recognize and normalize gene or phenotype names in text, in part due to our reliance on dictionary-based matching (17). For example, in an article (PMID:26937036), our method misses the association between the *miR-19b-3p* gene and *encephalitis* because this gene is not currently covered by UniProt/Swiss-Prot and thus was not retained into our gene name dictionary. On the other hand, in this specific article, we can see that the authors implicitly mention on *encephalitis* as `Japanese Encephalitis Virus-Mediated Inflammation' which indicates that authors do not always follow phenotype (or gene) name nomenclature while describing biomedical entities in their publications. In the future, we may consider machine learning approaches to overcome this limitation (33); however, the challenge is to use the background knowledge in ontologies as part of machine learning models (37). We observed false positive associations introduced by the abbreviations which are ambiguous with the gene names. For example, the term `GCL' is used as an abbreviation for `granular cell layer' as well as for Glutamate Cysteine Ligase. Furthermore, our approach relies on statistically significant associations between a gene and phenotype class, and the type of association is not considered; for example, whether a gene has a protective or causative relation to a phenotype cannot be detected by our method and can lead to further false positive associations.

We do not detect or consider negation that occurs in sentences; consequently, false positives may also be caused by considering co-occurrences between gene and phenotype mentions in sentences that express a negation. However, in our method we apply the NPMI measure to a large corpus; as long as co-occurrences between a gene and phenotype appear in negated form only in few sentences relative to how often they appear in non-negated sentences, they will not result in a significant co-occurrence.

While the statistical approach we use leads to more robust and generalized association results, it also has the limitation that our associations are extracted from an entire corpus and it is not easily possible to identify the specific sentence, abstract, or article that leads to an associations. We can, however, identify the set of all sentences in which a gene name and phenotype mention co-occur. Our results also indicate that the top-ranking associations resulting from our text-mining method can often be asserted directly as a gene–phenotype association, and most sentences that are used to generate the association can be used as evidence. In the future, we may further apply clustering to these sentences to reduce their number and make them more accessible.

## Conclusions

We developed a method that identifies gene–phenotype associations from the biomedical literature. Our method utilizes the semantics and structure of ontologies as background knowledge and performs a statistical analysis of co-occurrence relations between terms and phrases within a large text corpus. The impact of our method is twofold: first, we extracted and made available a set of candidate gene–phenotype associations that can serve as a foundation to improve manual curation of gene–phenotype associations, for example by suggestion candidate gene–phenotype pairs or suggesting associations that may have been missed; second, we have demonstrated that our associations can already improve computational analysis of phenotypes when investigating Mendelian diseases, and our results can therefore provide a set of electronically inferred annotations to include in certain types of computational analysis.

Our results are freely available (38) and will be updated frequently.

## Materials and Methods

### Ontologies and Resources used

We used the Open Access full text articles (http://europepmc.org/ftp/archive/v.2017.06/) (1.6 Million) from the Europe PMC database (39) as literature source. We used two comprehensive phenotype resources, HPO (3) (downloaded on 30/06/2017) and MP (14) (downloaded on 30/06/2017), and UniProt/Swiss-Prot (18) (downloaded on 1/May/2017) to identify phenotypes and gene classes in full text. We generated two dictionaries from the labels and synonyms in the resources for genes and phenotypes. We refined the dictionaries by filtering out terms which have less than three characters and terms that are ambiguous with common English words (e.g., `she' is a gene name) before applying text mining. Use of unrefined dictionaries would introduce potentially high numbers of false positives and therefore would reduce the system’s performance by affecting precision. Our final phenotype and gene dictionaries consisted of a total number of 48 122 (29 794 terms from MP, and 18 328 terms from HPO) and 142 310 distinct terms, respectively.

We used the gene–phenotype associations from HPO and MGI (downloaded on 30/07/2018) to analyze the overlapping associations between the two reference sets and the text mined extracts. Our dataset from HPO covers 99 333 gene–phenotype pairs while the dataset derived from MGI covers 211 012 gene–phenotype associations.

We used the PhenomeNET (7) ontology which includes the phenotypes from HPO and MGI to compute the semantic similarity between the genes and the diseases based on phenotypes.

We gathered disease–phenotype associations from the HPO database (downloaded on 30/07/2018). This dataset contains 88 103 disease–phenotype associations belonging to 7226 distinct diseases from OMIM.

We used gene–disease associations from OMIM and MGI in our experiments on recovering disease candidate genes (downloaded on 30/07/2018). The OMIM dataset covers 12 855 human gene–disease associations while the MGI dataset covers 8201 mouse gene–disease associations.

### Text mining gene–phenotype associations

We used WhatIzIt (17), a dictionary based named entity recognition tool, to annotate phenotype and gene mentions in full text articles. We used UniProt/Swiss-Prot (human and mouse genes only) to annotate gene names while we used MP and HPO to annotate phenotype names in publications.

We extracted gene–phenotype pairs based on their co-occurrences within sentences and use this information to determine whether there is a statistical association between a given gene and phenotype mention. We propagated the co-occurrence statistics through the phenotype ontologies (MP and HPO).

### Semantic Similarity

We used Resnik’s semantic similarity (25) to measure the similarities of phenotypes linked to genes and diseases. The similarity of two classes is formally defined as:

(2) \begin{equation*} sim(c_1, c_2)=\max_{c \in S(c_1, \ c_2)} - [\textrm{log}\ p(c)] \end{equation*}

where p(c) is the frequency with which c occurs within a set of entities (genes or diseases) annotated with classes from the same ontology. We used the Best Match Average (BMA) strategy to calculate the similarity between two sets of phenotypes:

(3) \begin{align*} sim_{BMA}(g_1,g_2)=\frac{\sum_{i=1}^{m}\max_1\leq\!_ j\! \leq_ n sim(c_{1i},c_{2j})+ \sum_{j=1}^{n}\max_1\leq\!_ i\! \leq_ n sim(c_{1i},c_{2j}) }{m+n}\end{align*}
